# Lentinan Combined with (125)I Brachytherapy for Recurrent Ovarian Cancer

**DOI:** 10.1155/2021/2472444

**Published:** 2021-11-09

**Authors:** Qin Jiang, Shuai Pang, Yifang Xia, Hongmei Sun, Yingying Yu

**Affiliations:** ^1^Department of Clinical Laboratory, Jiaozhou Central Hospital, Qingdao 266300, China; ^2^Department of Blood Transfusion, Yantai Yuhungding Hospital, Yantai 264000, China; ^3^Department of Imaging, Zhangqiu District People's Hospital, Jinan 250200, China; ^4^Hospital Infection-Control Department, Zhangqiu District People's Hospital, Jinan 250200, China; ^5^Department of Pharmacy, Qilu Hospital (Qingdao), Cheeloo College of Medicine, Shandong University, Qingdao 266035, China

## Abstract

**Objective:**

To investigate the clinical value of lentinan combined with (125)I brachytherapy in the treatment of recurrent ovarian cancer.

**Methods:**

A total of 160 patients with recurrent ovarian cancer admitted at Jiaozhou Central Hospital from June 2009 to October 2015 were enrolled in this study and randomly divided into observation group (80 cases) and control group (80 cases). The control group received chemotherapy. Observation group (80 cases) was treated with lentinan combined with (125)I brachytherapy on the basis of control group, and the efficacy, adverse reactions, and Karnofsky Performance Scale (KPS) and quality of life scale (QOL) scores of the two groups were analyzed and compared.

**Results:**

After treatment, the levels of CA125, CA199, and CA724 in the 2 groups were markedly lower than those before treatment, and the observation group was lower than the control group (*P* < 0.05). After treatment, the proportion of CD4^+^/CD8^+^ cells and helper T cells and NK cells in the control group remarkably depleted, while the proportion of CD4^+^/CD8^+^ cells, NK cells, and B cells in the observation group increased significantly compared to that before treatment, and the level of IgA, IgG, and IgM in the control group decreased, while that in the observation group showed no conspicuous difference compared with that before chemotherapy (*P* > 0.05). The effective rate of observation group (85%) was higher than that of control group (75%) (*P* < 0.05). The overall survival of patients in the control group was (16.2 ± 2.04) months and that of the observation group was (24.8 ± 1.8) months. KPS and QOL scores in both groups were enormously higher than those before treatment, and the observation group was higher than the control group (*P* < 0.05). The incidence of hemoglobin reduction, leukopenia, aglobulia, granulocytopenia, nausea and vomiting, hepatorenal toxicity, and neurovirulence in the observation group was significantly lower than that in the control group.

**Conclusion:**

Lentinan combined with (125)I brachytherapy is effective in treating recurrent ovarian cancer, with mild adverse reactions and good tolerance.

## 1. Introduction

Ovarian cancer (OC) accounts for 5% of cancer deaths in women [1]. Recurrent ovarian cancer refers to the signs of tumor recurrence in patients who have reached complete remission after optimal cytoreduction and adjuvant platinum-based chemotherapy and have to suspend the drug for half a year [2]. After relapse, first-line chemotherapy drugs such as topotecan and doxorubicin alone have no effect on prognosis, which triggers many adverse reactions, and most patients with relapse have a poor quality of life. Therefore, additional and alternative therapeutic strategies to improve outcomes are necessary.

In the last decade, percutaneous image-guided (125)I implantation has been regarded as a useful and minimally invasive treatment for various malignancies owing to its curative effects, such as head and neck tumors and pancreatic, lung, and hepatic cancer [3–6]. At the same time, its successful application in OC is still limited. Meanwhile, (125)I particle implantation can also cause a series of adverse reactions, such as pneumothorax, pulmonary hemorrhage, and sputum blood. New drugs are urgently needed to alleviate its side effects. Lentinus edodes (Shiitake) is the world's second-largest cultivated medicinal, edible fungus and has a long history in the oriental folklore of treating tumors [7]. Lentinan is the active ingredient extracted from the natural product Lentinus edodes mycelium, mainly composed of B1-3, B1-6 dextran [8]. Previous studies suggest that lentinan exerts its antitumor effects by activating the host's immune response instead of directly attacking cancer cells [9]. The antitumor activity of lentinan was believed to be mediated through a thymus-dependent immune mechanism.

In this study, the clinical study of lentinan combined with (125)I brachytherapy in patients with recurrent ovarian cancer was conducted to explore the application value of this therapeutic approach in such patients.

## 2. Methods

### 2.1. Patients

A total of 160 patients with recurrent ovarian cancer after surgery from June 2009 to October 2015 at the Jiaozhou Central Hospital, Qingdao, Shandong, China, were enrolled in this study. Biopsy pathology showed 70 cases of poorly differentiated serous papillary cystadenocarcinoma, 60 cases of moderately differentiated serous papillary cystadenocarcinoma, 14 cases of highly differentiated serous papillary cystadenocarcinoma, and 16 cases of poorly differentiated mucinous papillary cystadenocarcinoma, all of which were confirmed as recurrent oocyte carcinoma by imaging and histopathological examination. All patients underwent surgical treatment in the early stage, and the preoperative TNM staging was stage III-IV. The tumor was confirmed to be recurrent by ultrasound biopsy after surgery, and the time between the first operation and the current recurrence was 24.2 ± 2.3 months. Karnofsky Performance Scale (KPS) was >60, and estimated survival time was >6 months. According to the principle of randomness, 160 cases subjects were divided into 80 cases in the observation group and 80 cases in the control group. All patients signed informed consent before participation, and this study was approved by the Ethics Committee of the Jiaozhou Central Hospital, Qingdao, Shandong, China.

### 2.2. Study Design

The control group was intravenously injected with Taxol liposome (135 mg/m^2^). Before chemotherapy, dexamethasone and diphenhydramine were routinely pretreated. Then arterial infusion chemotherapy was performed the next day. The catheter was introduced through femoral artery puncture by the Seldinger method; under the monitoring of DSA, the catheter was selected to the inferior mesenteric artery or internal iliac artery for angiography to observe the blood supply status of the tumor. Carboplatin (200 mg/m^2^) was diluted and slowly injected into the tumor supplying artery through the catheter. Routine symptomatic treatment such as liver protection and antiemetic and gastric protection was given, repeated once every 21 days.

The observation group was treated with (125)I particle implantation and Lentinus edodes polysaccharide tablets. The number of (125)I seeds and the planning target volume (PTV) were determined using a treatment planning system. PTV was defined as tumor target area expansion of 5–10 mm, and matched peripheral dose (MPD) was 110–160 Gy. During the operation, CT scanning was used to locate the puncture direction and puncture point. After disinfection and anesthesia, (125)I seeds were implanted percutaneously under the guidance of CT with an interval of 0.5 cm. A postoperative CT review was performed to understand the particle distribution. When lesions were present in both ovaries, the interval of particle implantation in the other lesion should be more than 3 days. Lentinus edodes polysaccharide tablets (Kaifeng Pharmaceutical Co., Ltd., Chinese drug approval number H41025015) were orally administered with 1 tablet per time and 2 times per day for 2 months. Within 3 days after (125)I seed implantation, the same chemotherapy treatment was performed as the control group.

### 2.3. Testing Index

Venous blood was collected before and after treatment to separate the serum. Then the level of IgG, IgM, and IgA in serum was determined by immunoturbidimetry. The kit was provided by Changchun Huili Biotechnology Co., Ltd.

Total 3 mL of fasting peripheral blood was collected from all patients before and after treatment. Flow cytometry was performed to evaluate the immune function of patients in both groups within 1 week after treatment. Total T cells (CD3^+^), helper T cells (CD3^+^ CD4^+^), killer T cells (CD3^+^CD8^+^), CD4^+^/CD8^+^, and NK cells (CD3^−^CD56^+^), CIK cells (CD3^+^CD56^+^), regulatory T cells (CD4^+^ CD25high^+^ FOXP3^+^), CD 14^+^, and CD 19^+^ B cells were detected.

Laboratory tests including hemoglobin (HGB), white blood cells (WBC), platelets (PLT), neutrophils (NEU), red blood cells (RBC), aspartate aminotransferase (AST), alanine aminotransferase (ALT), creatinine (CREA), urea nitrogen (BUN), and electrocardiograph also were recorded before and after treatment. Chemotherapy side effects were graded according to WHO acute and subacute toxicity criteria, which were divided into 0, I, II, III, and IV. Blood toxicity, gastrointestinal reaction, liver and kidney function, cardiotoxicity, and peripheral neurotoxicity were assessed and compared between the two groups.

### 2.4. Evaluation Criteria for Short-Term Efficacy

Efficacy was evaluated according to RECIST criteria. Complete remission (CR) means complete disappearance of all target lesions. Partial response (PR) means reduction of the sum of the maximum diameters of target lesions ≥30%. Stable disease (SD) is the sum of the maximum diameter of the target lesions decreases without reaching PR or increases without reaching PD. Disease progression (PD) is an increase of ≥20% in the sum of the maximum diameters of the target lesions or the appearance of new lesions. The efficiency (RR) was calculated by CR + PR, while disease control rate (DCR) was calculated by CR + PR + SD.

The patient's functional status was assessed with a KPS score, and the higher the score, the better the patients' functional status. Quality of life scale (QOL) (SF-36) was performed to evaluate patients' quality of life, and the higher the score, the better the quality of life.

All objects were followed up for 5–46 months, and the overall survival of patients in the two groups was statistically compared.

### 2.5. Statistical Method

SPSS 21.0 statistical software was performed for data analysis. The data were expressed by $\bar{x}$ ± *s*. *χ*^2^ test or *t* test was applied for comparison between groups, Kaplan–Meier survival analysis and log-rank test univariate analysis were used to compare the difference in survival rates between the two groups. *P* < 0.05 indicates that the difference is statistically significant.

## 3. Results

### 3.1. Pathological Characteristics

There were 160 patients with recurrent ovarian cancer, including 80 cases in the control group and 80 cases in the observation group.  Table 1 shows that there were no significant differences in age, TNM stage, pathological type, differentiated status, and CA125 level at recurrence between the two groups, as shown in Table 1.

### 3.2. Blood-Related Indexes

The HGB, WBC, PLT, NEU, and RBC levels in two groups before treatment showed no statistically significant difference (*P* > 0.05). After treatment, the level of HGB, WBC, NEU, and RBC in control group decreased enormously, and the level of WBC and NEU in observation group attenuated tremendously, while the level of HGB, WBC, NEU, and RBC in observation group was obviously higher than that of control group, as shown in Table 2.

### 3.3. Immunologic Function

In order to further analyze the effect of Lentinus edodes polysaccharide combined with (125)I particle and chemotherapy treatment on the immune status of patients, the proportion of each immune cell in peripheral blood of patients before and after treatment was compared, as shown in Table 3. After chemotherapy treatment, the proportion of helper T cells, CD4^+^/CD8^+^ cells, and NK cells in the control group remarkably depleted, while after Lentinus edodes polysaccharide combined with (125)I particle and chemotherapy treatment, the proportion of CD4^+^/CD8^+^ cells, NK cells, and B cells in the observation group aggrandized enormously compared to that before treatment.

The level of immunoglobulin (IgA, IgG, and IgM) in the control group decreased immensely after chemotherapy compared with before chemotherapy. In contrast, the level of IgA, IgG, and IgM in the observation group showed no apparent difference after treatment compared with before chemotherapy (*P* > 0.05), as shown in Table 4.

### 3.4. Tumor Markers

There was no difference in the level of CA125, CA199, and CA724 between the two groups after treatment compared with before treatment (*P* > 0.05). After treatment, the levels of CA125, CA199, and CA724 in both groups alleviated broadly, while the levels of CA125, CA199, and CA724 in the observation group diminished prominently than the control group (*P* < 0.05), as shown in Table 5.

### 3.5. Therapeutic Effect

**Figure 1:**
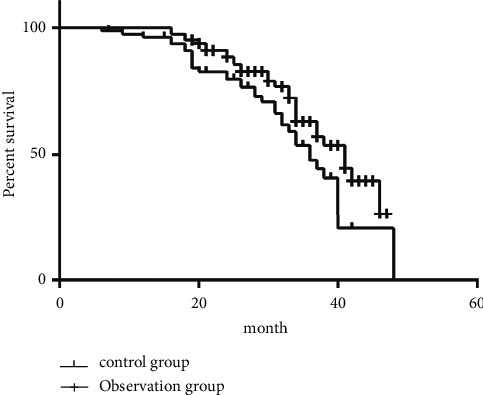
The overall survival of the two groups.

According to the evaluation of the therapeutic effect of solid tumor, the DCR of the two groups was 60/80 (75%) and 68/80 (85%), respectively, while the efficacy of Lentinus edodes polysaccharide combined with (125)I particle and chemotherapy treatment was superior to that of chemotherapy alone (*P* < 0.05) (Table 6). A total of 160 subjects were followed up for 5–46 months (median time: 26 months). The overall survival of patients in the control group was (16.2 ± 2.04) months and that of the observation group was (24.8 ± 1.8) months (Figure 1). Log-rank test was applied to compare the survival of the two groups, and the observation group was significantly higher than the control group.

### 3.6. KPS and QOL Score

There was no significant difference in KPS score and QOL score between the two groups before treatment. The scores of KPS and QOL in both groups were significantly improved after treatment, and the observation group was significantly higher than the control group (*P* < 0.05), as shown in Table 7.

### 3.7. Incidence of Adverse Reactions

The incidence of nausea and vomiting, neurotoxicity, and hepatorenal toxicity in the observation group was lower than that in the control group (*P* < 0.05), as shown in Table 8.

## 4. Discussion

Ovarian cancer is a gynecological malignant tumor with high incidence, and most patients are accompanied by ascites, which is difficult to treat and results in a poor prognosis. At present, chemotherapy is still the preferred treatment for recurrent ovarian cancer, but it is difficult to achieve the desired outcomes by chemotherapy or radiotherapy because of the large tumor load in patients with recurrent ovarian cancer [10]. Therefore, looking for adjuvant treatment options has become the focus of clinical research.

(125)I radioactive particles can have a continuous effect on tumor cells, block cells in *G*_2_/*M* phase, prolong cell cycle, and lead to the loss of proliferation ability of tumor cells [11, 12]. (125)I radioactive particles are implanted within the range of radiation therapy, and the dose of radiation dose decreases with the increase of distance. Therefore, (125)I particle implantation can produce a high dose at the site, while the radiation dose of surrounding normal tissues is low, with highly conformal radiation [13–15]. Zhao et al. found that (125)I seed implantation based on CT-guided 3D template-assisted technique can effectively alleviate the tumor and improve the survival rate in patients with refractory malignant tumors [16]. Despite the significant effect of (125)I seed implantation in the treatment of cancer, the adverse reactions caused by it cannot be ignored [17]. Zhang et al. reported that (125)I brachytherapy triggers a higher relative risk of pneumothorax, pneumorrhagia, and bloody sputum compared with chemotherapy alone [18]. Martinez-Monge et al. reported that the complication rate of 125I radiotherapy with short-range and high dose rate was 20% [19]. Therefore, it is equally important to kill tumor cells and protect immune function in tumor treatment.

Lentinan has significant inhibitory effects on the transplanted tumor, allogeneic tumor, and primary tumor, preventing carcinogenic effects caused by chemical factors or viruses, inhibiting and preventing postoperative micrometastasis of gastric cancer, lung cancer, liver cancer, rectal cancer, and breast cancer [20–24]. Kimura et al. used lentinan in combination with chemotherapy to treat recurrent gastric cancer and found that lentinan can reduce adverse reactions to chemotherapy and improve the immune and nutritional status of patients [25]. The mechanism of lentinan's action does not involve the direct attack on the pathogenic source, but to improve the balance of the host body by stimulating the maturation, differentiation and proliferation of immune cells, to restore and improve the responsiveness of host cells to lymphokines, hormones, and other physiologically active factors [26]. In this study, lentinan tablets combined with (125)I particle implantation were applied to observe the effects in antitumor and immune function.

In this study, we used lentinan combined with (125)I particle implantation to treat patients with recurrent ovarian cancer, and we found that the complete remission rate was 20% and partial remission rate was 60% after treatment, significantly higher than the control group, and the overall survival in the observation group was significantly higher than that in the control group, suggesting that (125)I particle implantation was effective in the treatment of recurrent ovarian cancer patients. After treatment, the proportion of CD4^+^/CD8^+^ cells, helper T cells, and NK cells in the control group remarkably depleted, while the proportion of CD4^+^/CD8^+^ cells, NK cells, and B cells in the observation group aggrandized enormously, and the level of IgA, IgG, and IgM in the control group decreased immensely, while that in the observation group did not change significantly, suggesting that lentinan can substantially improve the immunosuppression induced by radiotherapy and chemotherapy. The observation of toxicity in this study suggested that hematological toxicity in the control group mainly manifested as hemoglobin reduction, leukopenia, aglobulia, and granulocytopenia. At the same time, the above reactions were alleviated in the observation group after taking lentinan, indicating that lentinan can promote bone marrow hyperplasia. The main toxic reactions of the digestive system were nausea and vomiting; these reactions in this study mainly occurred 2–8 days after intravenous infusion, while these symptoms were relieved after lentinan treatment. Concurrently, the incidence of hepatorenal toxicity and neurovirulence could be alleviated by lentinan treatment.

In this comparative evaluation, we observed that the lentinan combined (125)I particles implantation treatment of recurrent ovarian cancer could improve the recent curative effect of recurrent ovarian cancer, strengthen the immune function of the patients, and reduce the treatment of gastrointestinal reaction and bone marrow suppression. It could lessen other side effects and improve the quality of life of patients, suggesting that lentinan combined (125)I brachytherapy may be an effective therapeutic approach for recurrent ovarian cancer.

## Figures and Tables

**Table 1:** Comparison of pathological characteristics between the two groups.

	Control group	Observation group	*P* value
Age	49 ± 5.3	48 ± 4.6	0.945
TNM stage			0.564
III	44 (55%)	47 (59%)	
IV	36 (45%)	33 (41%)	
Pathological type			0.215
Serous papillary cystadenocarcinoma	69 (86%)	75 (94%)	
Mucinous papillary cystadenocarcinoma	11 (14%)	5 (6%)	
Differentiated			0.341
Poorly differentiated	46 (58%)	40 (50%)	
Moderately differentiated	28 (35%)	32 (40%)	
Highly differentiated	6 (7%)	8 (10%)	
CA125			0.645
≤35 U/ml	21 (26)	25 (31)	
>35 U/ml	59 (74)	55 (69)	

**Table 2:** Comparison of blood-related indexes between two groups ($\bar{x}$ ± *s*).

	Control group		*P* value	Observation group		*P* value
	Before treatment	After treatment		Before treatment	After treatment	
WBC (×10^9^L)	7.02 ± 0.56	1.84 ± 0.24	<0.001	7.09 ± 0.48	4.05 ± 0.16^*∗∗*^	0.032
NEU (×10^9^L)	5.02 ± 0.45	1.45 ± 0.26	0.015	4.97 ± 0.78	2.68 ± 0.45^*∗*^	0.045
HGB (g/L)	115.84 ± 13.65	71.26 ± 10.23	0.002	116 ± 14.62	110.25 ± 12.15^*∗∗*^	0.315
PLT (×10^9^L)	251.23 ± 32.15	227.16 ± 26.12	0.124	249.13 ± 14.26	239.45 ± 32.16	0.421
RBC (×10^12^L)	4.87 ± 0.15	1.38 ± 0.24	0.026	4.97 ± 0.12	3.15 ± 0.21^*∗*^	0.051

Versus control group, ^*∗*^*P* < 0.05, ^*∗∗*^*P* < 0.01.

**Table 3:** Proportion of peripheral blood immune cells in the two groups before and after treatment (median (P25, P75)).

	Control group (%)		*P* value	Observation group (%)		*P* value
	Before treatment	After treatment		Before treatment	After treatment	
CD3^+^	75.76 (47.78, 87.79)	75.18 (52.36, 82.31)	0.915	74.54 (51.23, 80.31)	73.61 (50.31, 81.24)	0.769
CD3^+^ CD4^+^	48.89 (18.97, 65.12)	39.6 (19.02, 78.68)	0.034	45.36 (17.65, 71.62)	49.75 (13.64, 74.16)	0.054
CD3^+^CD8^+^	33.89 (17.84, 45.26)	35.62 (20.69, 49.74)	0.549	36.16 (21.36, 48.37)	34.15 (19.65, 47.59)	0.512
CD4^+^/CD8^+^	1.65 (0.61, 2.89)	1.09 (0.48, 2.84)	0.048	1.69 (0.69, 2.79)	2.43 (0.64, 3.86)^*∗*^	0.019
CD3^−^CD56^+^	9.65 (5.24, 41.26)	8.25 (5.81, 37.64)	0.031	9.26 (6.31, 42.36)	14.26 (5.96, 36.16)^*∗*^	0.023
CD3^+^CD56^+^	3.68 (0.42, 10.29)	3.45 (0.59, 9.78)	0.794	3.78 (0.39, 11.29)	4.22 (0.57, 9.78)	0.145
CD 14^+^	4.78 (0.23, 32.04)	5.21 (0.02, 24.17)	0.359	5.61 (0.29, 36.19)	6.26 (0.16, 30.26)	0.278
CD 19^+^	8.41 (2.71, 17.41)	8.14 (5.78, 37.26)	0.571	7.89 (2.61, 26.45)	11.45 (5.14, 33.15)^*∗*^	0.036
CD4^+^ CD25high^+^ FOXP3^+^	4.74 (0.04, 11.68)	3.36 (0.15, 12.27)	0.401	4.16 (0.12, 12.31)	5.48 (0.34, 16.45)	0.141

Versus control group, ^*∗*^*P* < 0.05, ^*∗∗*^*P* < 0.01.

**Table 4:** Comparison of immunoglobulin levels between the two groups ($\bar{x}$ ± *s*, g/L).

	Control group		*P* value	Observation group		*P* value
	Before treatment	After treatment		Before treatment	After treatment	
IgG	12.94 ± 1.02	10.84 ± 0.24	0.025	12.77 ± 0.93	12.65 ± 1.02^*∗*^	0.082
IgM	1.76 ± 0.21	1.20 ± 0.12	0.004	1.75 ± 0.36	1.88 ± 0.36^*∗∗*^	0.056
IgA	2.39 ± 0.23	1.87 ± 0.36	0.016	2.31 ± 1.01	2.44 ± 0.95^*∗*^	0.054

Versus control group, ^*∗*^*P* < 0.05, ^*∗∗*^*P* < 0.01.

**Table 5:** Comparison of tumor markers in blood between the two groups before and after treatment ($\bar{x}$ ± *s*).

Group	CA125 (U/ml)		*P* value	CA199 (IU/ml)		*P* value	CA724 (IU/ml)		*P* value
	Before treatment	After treatment		Before treatment	After treatment		Before treatment	After treatment	
Control group	48.32 ± 12.32	25.64 ± 6.15	0.012	81.23 ± 7.36	53.21 ± 3.64	0.004	34.68 ± 4.61	14.56 ± 4.8	0.006
Observation group	49.12 ± 11.13	18.45 ± 3.14	0.003	80.64 ± 6.45	41.54 ± 4.65	<0.001	35.48 ± 6.48	9.12 ± 4.71	<0.001
*P* value	0.821	0.027		0.754	0.018		0.634	0.035	

**Table 6:** Comparison of short-term efficacy between the two groups, *n* (%).

Group	*n*	CR	PR	SD	PD	RR	DCR
Control group	80	13 (16%)	45 (56%)	2 (3%)	20 (25%)	58 (73%)	60 (75%)
Observation group	80	16 (20%)	48 (60%)	4 (5%)	12 (15%)	64 (80%)	68 (85%)
*χ* ^2^	4.312						
*P* value	0.026						

**Table 7:** Comparison of KPS and QOL scores between the two groups ($\bar{x}$ ± *s*).

Group	KPS score		*P* value	QOL score		*P* value
	Before treatment	After treatment		Before treatment	After treatment	
Control group	71.43 ± 5.31	80.12 ± 6.32	0.034	24.61 ± 6.21	37.65 ± 5.87	0.041
Observation group	70.65 ± 5.87	93.12 ± 5.87	0.005	25.65 ± 6.48	50.21 ± 4.87	0.002
*P* value	0.742	0.025		0.674	0.035	

**Table 8:** Comparison of the incidence of adverse reactions between the two groups (*n*).

Adverse reactions	Control group				Observation group				*P* value	*χ* ^2^
	I	II	III	IV	I	II	III	IV		
Nausea and vomiting	16	8	2	0	7	2	0	0	0.005	5.123
Renal injury	5	0	0	0	1	0	0	0	0.041	2.654
Liver injury	6	1	0	0	2	0	0	0	0.036	4.659
Neurovirulence	4	0	0	0	3	0	0	0	0.157	1.054

## Data Availability

The data used to support the findings of this study are available from the corresponding author upon request.
